# Supplementary material for: Splice-Junction-Based Mapping of Alternative Isoforms in the Human Proteome
Source: Cell Rep. Author manuscript; Available in PMC 2020 Jan 15. (PMC6961840; doi:10.1016/j.celrep.2019.11.026)
Supplement: 2 [file NIHMS1546469-supplement-2.zip › DF1/PXD010154/Esophagus/Esophagus_11_PKM_DQEAPPHSGTAFIQTQQLHAAMADTFLEHMCR.pdf]

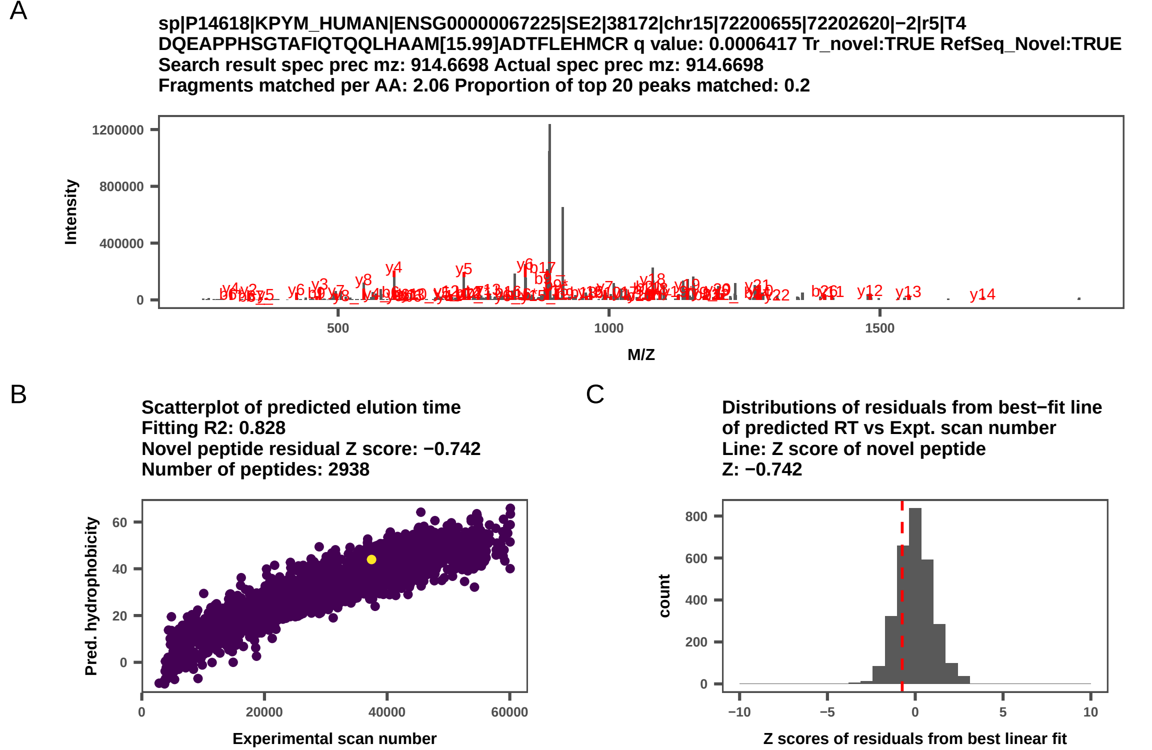
A
sp|P14618|KPYM_HUMAN|ENSG00000067225|SE2|38172|chr15|72200655|72202620|−2|r5|T4
DQEAPPHSGTAFIQTQQLHAAM[15.99]ADTFLEHMCR q value: 0.0006417 Tr_novel:TRUE RefSeq_Novel:TRUE
Search result spec prec mz: 914.6698 Actual spec prec mz: 914.6698
Fragments matched per AA: 2.06 Proportion of top 20 peaks matched: 0.2
Intensity
1200000
800000
400000
0
y4
y5
y6
y8
y12
y13
y14
M/Z
500
1000
1500
B
Scatterplot of predicted elution time
Fitting R2: 0.828
Novel peptide residual Z score: −0.742
Number of peptides: 2938
Pred. hydrophobicity
60
40
20
0
0
20000
40000
60000
Experimental scan number
C
Distributions of residuals from best−fit line
of predicted RT vs Expt. scan number
Line: Z score of novel peptide
Z: −0.742
count
800
600
400
200
0
−10
−5
0
5
10
Z scores of residuals from best linear fit
